# The Ideal Canine Companion: Re-Exploring Australian Perspectives on Ideal Characteristics for Companion Dogs

**DOI:** 10.3390/ani14243627

**Published:** 2024-12-16

**Authors:** Emma S. Power, Jessica Dawson, Pauleen C. Bennett

**Affiliations:** Anthrozoology Research Group, School of Psychology and Public Health, La Trobe University, Bendigo, VIC 3552, Australia; 19325443@students.latrobe.edu.au (E.S.P.); jessica.dawson@latrobe.edu.au (J.D.)

**Keywords:** ideal dog, human–animal relationship, behaviour, companion, anthrozoology

## Abstract

Defining the ideal characteristics of canine companions is imperative to those seeking to improve human–dog relationships. Previous investigations into the preferred traits of companion dogs were conducted before substantial recent events, such as the rise of social media and the COVID-19 pandemic, which altered human lifestyles and potentially affected canine preferences and owner satisfaction. This study surveyed Australian adults (*n* = 337) online to assess these shifts. The ideal dog was characterised as medium-sized, with a short- low- or non-shedding coat, and acquired as a puppy from a shelter/rescue. Desired traits included being affectionate, healthy, non-aggressive, safe with children, housetrained, non-destructive when alone, and not prone to escaping. Principal Component Analysis identified five factors: ‘calm and obedient’, ‘inhibitory control’, ‘energy and drive’, ‘non-aggressive and safe’, and ‘affectionate and healthy’. Demographic variables of age, gender, perceived income, and household children influenced preferences. No significant differences in owner satisfaction with behaviour were observed between dogs acquired during versus outside the pandemic. The findings align with prior Australian research, suggesting enduring stability in preferences for the ideal companion dog. These insights offer valuable guidance for breeders, prospective owners, and policymakers to reduce dog relinquishment and improve human–dog relationships.

## Simple Summary

What makes ‘man’s best friend’ a human’s ideal friend? Previous studies examining ideal characteristics for companion dogs were conducted before the rise of social media and the COVID-19 pandemic, events that changed how humans live and, potentially, their preferences for the ideal dog. Over 330 Australian adults were recruited online and surveyed. The ideal dog was found to be medium-sized, with a short low- or non-shedding coat. It would be acquired as a puppy from a shelter/rescue. It would also be affectionate, healthy, non-aggressive, safe with children, housetrained, non-destructive when left alone, and would not escape the property. Differences across age, gender, perceived income in comparison to others, and number of household children were observed for desired physical and behavioural traits, but no significant differences regarding dog behaviour satisfaction were found between pandemic and non-pandemic acquisitions. Findings resemble those of a previous Australian ideal dog study, suggesting that preferences for the ideal dog are relatively stable. Breeders, prospective owners, and policymakers can benefit from this research by using the findings to guide choices, reduce relinquishment, and improve human–dog relationships.

## 1. Introduction

Humans and dogs (*Canis lupus familiaris*) have coexisted for millennia, with domestication of the wolf (*Canis lupus*) commencing around 39,000 years ago, marking the beginning of a long and intertwined history [1]. Artificial selection for specific purposes started 3–4000 years ago [2], with most breeds emerging in the last two centuries [3]. While distinct breeds were initially developed for specific functional roles, dogs are now primarily kept for companionship [2,4,5]. However, many retain physical and behavioural traits linked to their original purposes, raising concerns about their compatibility with modern lifestyles. Behavioural issues, such as hyperactivity, anxiety, and aggression, are among the leading causes of dog relinquishment [4,5,6,7,8], along with unrealistic owner expectations and misconceptions about breed requirements [8,9,10,11].

Previous studies on ideal dogs conducted more than a decade ago found preferences for friendly, obedient, and child-safe dogs that were affectionate, housetrained, came when called, and were unlikely to escape [2,6]. Behavioural traits were prioritised over physical features, although participants also favoured medium-sized dogs with short, straight hair [2,6]. Consistent with some of these preferences, recent pet surveys found that the most popular breeds in Victoria, Australia, included Cavoodles (Cavalier King Charles Spaniel X Poodle), Labrador Retrievers, Greyhounds, Border Collies, and Golden Retrievers [12]. Nationwide, Labrador Retrievers, Staffordshire Bull Terriers, Golden Retrievers, Jack Russell Terriers, and German Shepherd Dogs were popular [13]. Not all of these popular breeds align with the previously identified ideal preferences. Additionally, Poodle crosses, such as Cavoodles, were rare during the previous studies but now make up approximately 20% of dogs purchased in Australia [14,15,16]. This suggests that some preferences may have changed and were potentially influenced by recent societal changes.

One major societal change has been a rise in the impact of social media. When King and colleagues [2] first reported on Australian ideal dog characteristics in 2009, social media was in its infancy. However, visual-based platforms like Instagram and TikTok have since given rise to influencers [17] and monetised pet accounts [18,19]. Rooted in evolutionary biology, humans have an innate desire to fit in [14,20]. This influences various preferences, from fashion to voting [21,22]. Dog breed preferences are no exception, as seen by the 424% increase in Dalmatian registrations in the United States following the release of Disney’s 101 Dalmatians in 1985 [21]. Social media provides global access to lifestyles that others often aspire to emulate [14], and this visibility has contributed to trends in dog breed popularity [7,14,23], with veterinarians acknowledging the strong impact of social media on owner choices [14]. 

While social media drives interests, it also carries risks. Many trending breeds, such as the brachycephalic (flat-faced) French Bulldog, may have significant health and welfare issues [9,22], while others marketed as ‘hypoallergenic’ often require extensive regular grooming [15,16,22]. Audiences may be attracted to these breeds due to perceiving them as toy-like or infantile-looking [9,24], but the curated portrayal of pets on social media can mislead consumers and may cause them to underestimate the associated care demands. Moreover, studies show that some popular breeds are more prone to inherited disorders and behavioural problems, including owner-directed aggression, fear of other dogs, and separation-related issues [25]. These issues could potentially strain the human–dog relationship, but they are rarely visible when celebrities and other influencers share photos and anecdotes about their pets. 

A second major societal change in recent years was the COVID-19 pandemic. This caused unprecedented lifestyle changes, including remote working, restricted movement, and social isolation, leading to a drastic surge in mental health issues and loneliness [10,26] and putting a significant strain on the healthcare system. Many households added new pets during this time, with almost 2 million pets acquired during extensive lockdowns in Australia [13]. Before the pandemic, dog ownership had remained relatively steady for at least a decade, at around 40% of households [2]. This rose to 47% in 2021, which equated to approximately 6.3 million pet dogs in Australia, with 55% aged under five years [13]. A similar global rise in dog acquisitions became known as the ‘Pandemic Puppy Phenomenon’ [10,13,26,27].

Many pandemic-acquired dogs have since been surrendered to shelters, often due to perceived behavioural problems, posing a welfare issue for dogs [26] and negatively impacting shelter workers [28]. Contributing factors may include impulsive purchasing without proper research or planning regarding the breed and lack of knowledge about dog care requirements. Strong demand also meant that dogs may have been purchased without due consideration of the sire, dam, and puppy’s health and living conditions [10], which can impact a puppy’s health, and fuel poor breeding conditions, jeopardising animal welfare. The Kennel Club [29] found that one in four puppies in the United Kingdom was acquired impulsively during the pandemic. First-time owners were also more likely to acquire a pet [26], accounting for nearly half of new pets in Australia [13]. These novice owners lack experience and may have unrealistic expectations [10]. Lockdowns also limited access to training classes and socialisation opportunities, potentially exacerbating behavioural problems [26]. Although lockdowns initially provided ample time to bond with new pets and motivation to exercise outdoors [10,13], returning to pre-pandemic routines introduced new challenges, such as separation anxiety [30]. The pandemic may have shaped preferences for characteristics perceived as more conducive to the companionship role, such as non-shedding, smaller, human-oriented breeds [22,31], but these characteristics may be less suited to owners’ busier, post-pandemic lifestyles, whereby owners may have less time to dedicate to their dogs’ needs and requirements. Thus, the pandemic may have reshaped perceptions of the ideal dog in ways not conducive to optimal long-term outcomes. 

Because humans and canines benefit considerably from close and affectionate relationships [32], understanding ideal dog characteristics is critical for improving owner satisfaction and dog welfare. It has been fifteen years since the previous Australian ‘ideal’ dog study was conducted [2] and, since then, unprecedented societal changes have occurred. Therefore, the current study aimed to re-investigate what behavioural and physical characteristics Australians prefer in their companion dogs, their ideal management requirements, and whether demographics influence these preferences. The study also sought to gather information on participants’ actual dog characteristics as a descriptive comparison to the ideal traits and to determine if acquisition during the pandemic is associated with owner satisfaction with dog behaviour. 

## 2. Materials and Methods

### 2.1. Participants

A sample of 343 Australian participants was recruited online through Prolific Academic Ltd. (www.prolific.com; accessed July 2024). This is an online platform based in London, UK, and first released in 2014, whereby registered participants can be recruited for research in exchange for a small payment. Six responses were discarded for incomplete data. Inclusion criteria required participants to be current Australian residents, at least 18 years of age, and fluent in English. Both dog owners and non-owners were invited to participate. 

### 2.2. Questionnaire

The original questionnaire developed by King et al. [2] was modified to reflect contemporary language, societal changes, and the aims of the present study. Key updates included adding detailed questions on acquisition sources and motivations, costs associated with dog management, expanded information about physical traits, and pandemic-related factors. We also adjusted costs for inflation, removed irrelevant or potentially confusing options, and adjusted language to be more gender inclusive. The resulting survey was hosted on REDCap (projectredcap.org), a secure online electronic data capture tool hosted at La Trobe University, and consisted of six sections. It took approximately 15 min to complete. The full survey is available in Supplementary Materials File S1 and the original survey in Supplementary Materials File S3. 

Section A consisted of 7 questions and sub-questions concerning the participants’ ideal dog acquisition preferences (amount of thought, source, age at acquisition) that were presented on 5-point scales. Section B examined the costs (financial and time dedication) associated with the ideal dog. Five response options were provided for each question. Section C consisted of 41 statements regarding ideal dog behaviours. Participants were asked to rate the importance of these statements on a 5-point Likert-type scale, which ranged from extremely unimportant (1) to extremely important (5). Section D covered the physical characteristics (sex, neutering, size, breed type, coat colour, length, texture) of the ideal dog. Most of the 16 items provided five response options regarding preferences; there were also open-ended questions asking participants for their preferred breed or breed types and questions regarding the importance of factors relating to different breed types, with a 5-point Likert-type scale ranging from extremely unimportant (1) to extremely important (5). Section E comprised 22 items asking dog-owning participants about the characteristics of their current or previous dog. These questions resembled those in Sections A, B, and D. Section F consisted of 19 questions that collected participants’ demographic and lifestyle information. 

### 2.3. Procedure

The La Trobe Human Research Ethics Committee granted ethical approval for the study (HEC24213). All data were collected between 25 July and 31 July 2024. Participants’ personal Prolific profiles were automatically filtered based on the inclusion criteria. Those eligible to participate received an automated invitation, and those who wished to participate were directed to the survey site to complete the questionnaire. Informed consent was obtained, and participants entered their Prolific identification number for reimbursement, which they received once they finished and were presented with a hyperlink to the study completion page on Prolific. 

### 2.4. Statistical Analysis

The raw survey data were exported from REDCap into Microsoft Excel for manual cleaning, following Pallant’s [33] instructions. Participants were removed from the data set if the full survey was incomplete. The data were then analysed using Jamovi statistical software (version 2.3). 

#### 2.4.1. Describing the Ideal Dog 

Descriptive statistics were obtained to summarise the data set. Principal Component Analysis (PCA) with an oblimin rotation condensed behavioural items from Section C. Preliminary checks included Kaiser–Meyer–Olkin (KMO) and Bartlett’s Test of Sphericity. The number of factors was determined based on eigenvalues, parallel analysis, and scree plot consideration, following Pallant’s [33] recommendations. Five factors with good fit were identified. Items loading onto each factor were summed, and each participant’s mean score on each factor was calculated. 

#### 2.4.2. Demographics’ Influence on Ideal Traits

Spearman’s correlations, Mann–Whitney U tests, and chi-square analyses were used to determine associations between demographic factors and ideal physical and behavioural dog preferences. Chi-square examined categorical variables, with Bonferroni adjustments where necessary. Correlations were conducted where continuous or ordinal variables were being assessed, with a comparison-wise α set at 0.00167 to compensate for the large number of correlations as per a Bonferroni calculation. Mann–Whitney U tests replaced t-tests for dichotomous and continuous or ordinal variables due to violations of normality and homogeneity, with Bonferroni adjustments controlling for Type I error. 

#### 2.4.3. Participants Actual Dogs

Descriptive statistics were used to summarise the data set. 

#### 2.4.4. Owner Satisfaction with Dog Behaviour and the COVID-19 Pandemic

Assumptions of normality were violated, so a Kruskal–Wallis was used in place of a one-way ANOVA to test for differences in behavioural satisfaction based on acquisition timing—pre-pandemic (before 2020), during pandemic (2020 and 2021), and post-pandemic (after 2021). 

## 3. Results

### 3.1. Sample Demographics

Participants ranged from 18 to 84 years (M = 35, SD = 12), with most born in Australia (74.6%). Of the 337 participants, 52.9% identified as female and 43.5% as male. Nearly half (46.3%) of the respondents resided in households containing two adults, and the majority had no children at home (70.3%). Approximately two-thirds (69.7%) were current or previous dog owners (Table 1). 

### 3.2. Ideal Dog: Physical Characteristics, Acquisition, and Maintenance 

Descriptive statistics are available in Supplementary Materials File S2 and are summarised below. Most participants (63.5%) did not have a preferred sex for their ideal dog, but female dogs were preferred by 20.7% and male dogs by 15.7%. De-sexed (spayed/neutered) dogs were preferred by 79.4%, with 2.4% preferring a sexually intact dog. Participants mostly showed no preference regarding whether their dog was purebred or not (73%), with 19.5% preferring purebred dogs and 7.4% preferring mixed breed or designer (purposefully cross-bred) dogs. A short coat length was preferred by 39.8% of participants, with medium length preferred by 30.3%. Approximately half of the sample had no preference for coat type (51.3%), while 35.8% preferred smooth-coated dogs. Regarding shedding level, many respondents preferred a low- (56.7%) or non-shedding (26.6%) dog, whereas 11.9% indicated no preference. Preferred sizes included medium (11–20 kg; 52.2%), small (3–10 kg; 27.9%), and large (21–40 kg; 16.6%).

Over 35% of participants (37.6%) showed no preference regarding whether their ideal dog was acquired directly from a breeder, with 32.5% saying they would prefer not to acquire their dog from a breeder and 29.9% saying they would prefer to do so. Acquisition from a shelter or rescue was preferred by 57.9%, with 23.1% reporting no preference regarding acquiring their dog from a shelter or rescue organisation and 19% preferring not to obtain it from this source. Cost expectations varied, with 40.2% preferring to spend less than AUD 1000 and 29.2% between AUD 1000 and AUD 2000. The majority of participants (68.2%) preferred to acquire their ideal dog as a puppy, with 8.6% preferring to acquire an adult dog and 23.1% expressing no preference. Participants who preferred a puppy reported that their ability to form stronger bonds (92.6%), cuteness (87%), trainability (80.8%), and having more time with the dog (70%) were the main reasons for this preference. The ability to choose a breeder (59.6%) and the availability of a particular breed (59.1%) were also important. Those who preferred to acquire their ideal dog as an adult rated it having already had basic training (75.8%), avoiding the puppy stage (72.4%), and a more visible personality (72.4%) as the most important reasons behind their preferences, with altruism (51.7%) and a lower cost (44.8%) also being influential. 

Participants were also asked about preferences regarding the cost of maintenance and their preferred time spent exercising and grooming their ideal dog. Australian currency was used; at the time of the study 1 AUD = USD 0.65. Half of the participants (50%) were willing to pay AUD 41–80 a week to maintain their dog, while 31% indicated an ideal budget of AUD 20–40. Almost half of the sample (48.1%) were willing to dedicate 31 to 60 min daily to exercising their ideal dog, whereas 37.8% were willing to spend 16 to 30 min. On the other hand, 39.8% were willing to spend 1 to 15 min a week grooming their ideal dog, with 36.5% willing to spend 16 to 30 min. 

### 3.3. Behavioural Characteristics of the Ideal Dog

On five-point scales ranging from (1) extremely unimportant to (5) extremely important, mean ratings for various behaviours ranged from 1.7 to 4.65, with the highest-valued traits being ‘My ideal dog is not destructive when left alone for long periods’ (M = 4.65, SD = 0.62), ‘My ideal dog is physically healthy’ (M = 4.59, SD = 0.61), ‘My ideal dog is fully housetrained’ (M = 4.58, SD = 0.66), ‘My ideal dog does not escape from my property’ (M = 4.56, SD = 0.64), and ‘My ideal dog is safe with children’ (M = 4.54, SD = 0.73). From the results of the PCA, five behavioural factors were created, ‘Calm and obedient’, ‘inhibitory control’, energy and drive’, ‘non-aggressive and safe’, and ‘affectionate and healthy’. Descriptive statistics for these factors are included in Table 2. 

Using PCA, the 41 behavioural statements were reduced to five factors that explained 50.5% of the total variance, with 32 out of 41 items retained and good reliability (Cronbach’s alphas ranged from 0.70 to 0.83; Table 2). The first factor, termed ‘calm and obedient’, included behaviours related to the dog being calm and coming when called. Factor 2, ‘inhibitory control’, comprising natural dog behaviours, often undesired by owners, such as eating faeces and sexual behaviours. The third factor, ‘energy and drive’, consisted of items related to the dog’s energy, attentiveness, hunting, and protective instincts. Factor four, ‘non-aggressive and safe’, included being safe with children and not showing aggression towards humans or other dogs. The final factor, ‘affectionate and healthy’, encompassed friendly behaviours and living a long, healthy life. The ‘affectionate and healthy’ and ‘non-aggressive and safe’ factors received the highest mean scores (M = 4.32, SD = 0.50 and M = 4.22, SD = 0.55, respectively), indicating that behaviours relating to these subscales may be viewed with higher importance than those in the other factors. In contrast, ‘energy and drive’ behaviours may be considered less important than other subscales.

### 3.4. Associations Between Demographics and Ideal Dog Characteristics

Table 3 shows Spearman’s correlation analyses. Because of the large number of correlations conducted, Bonferroni adjustments were adopted to reduce the likelihood of Type I errors. With a revised alpha value of 0.00167, very few correlations were significant. Mann–Whitney U outputs are available in Supplementary Material File S2. 

#### 3.4.1. Gender

Female participants more strongly preferred their ideal dog to be de-sexed than did males, although most males also strongly preferred a de-sexed dog (χ^2^(1) = 7.18, *p* = 0.007). Females also rated ‘calm and obedient’ (*U*(308) = 9292, *p* < 0.001) and ‘affectionate and healthy’ (*U*(315) = 9845, *p* = 0.001) behaviours to be significantly more important than did males. Small differences were observed for ideal size, ‘inhibitory control’, and ‘non-aggressive and safe’ behaviours, but these were not significant. 

#### 3.4.2. Age

Age was positively correlated with the amount of time participants would be willing to exercise their ideal dog (ρ(335) = 0.21, *p* < 0.001), as well as with ‘calm and obedient’ (ρ(324) = 0.02, *p* < 0.001) and ‘non-aggressive and safe’ (ρ(325) = 0.21, *p* < 0.001) behavioural subscales. 

#### 3.4.3. Ownership

Small differences between participants who were current dog owners and those who were not were observed for the ideal time spent exercising and time spent grooming; however, these were not significant at the conservative level employed. 

#### 3.4.4. Income

Participants’ perceived income compared to others in the community was positively correlated with the cost of acquiring the ideal dog (ρ(333) = 0.20, *p* < 0.001). 

#### 3.4.5. Household Children

Households without children were more likely to prefer acquiring their ideal dog from a shelter or rescue organisation (χ^2^(1) = 8.72, *p* = 0.003). No significant correlations were observed at α = 0.00167, although several associations approached significance.

### 3.5. Participants’ Actual Dog Characteristics

Full descriptive data are available in Supplementary Materials File S2. Of the respondents, 45.1% were very happy with their current or previous dog’s behaviour, and 43% were very happy with their health and longevity. The sex distribution of participants’ current or previous dogs was 51.9% male and 47.7% female; the remaining 0.4% could not remember. Dogs were medium-sized (11–20 kg; 37.4%), small (3–10 kg; 29.4%), and large (21–40 kg; 26.4%). The majority were purebred (49.6%) or mixed (44.4%), with designer-bred being 6%. Shedding levels for actual dogs were low (44.1%) or moderate (32.3%), with non-shedding being 12.7% and heavy shedding 10.9%. 

Most dogs had been acquired under 3 months of age (41.5%) or between 3 and 6 months (32.3%), with 11.8% being acquired between 7 and 12 months, 7.9% between 13 months and 3 years, and 6.6% at over 3 years of age. Participants had acquired their dogs from breeders (35.5%), friends or family (26.3%), a shelter or rescue organisation (19.3%), from a pet shop (10.5%), or from other means (8.3%). Many were acquired for less than AUD 1000 (38.5%), whereas other respondents spent nothing (21.5%), between AUD 2001 and AUD 4,000 (16.7%), between AUD 1001 and AUD 2000 (15.4%), and between AUD 4001 and AUD 10,000 (2.2%); 6.1% could not remember or did not know. 

Many participants (43.9%), on average, spent AUD 20-40 a week maintaining their dog, 33.3% spent between AUD 41 and AUD 80, 13.6% spent under AUD 20, 6.6% between AUD 81 and AUD 150, and 2.6% spent more than AUD 150. Dog owners in the sample tended to spend between 16 and 30 min a day exercising their dog (42.5%), while 29.8% spent 31–60 min, 15.4% spent 1–15 min, 11% spent more than 60 min, and 1.3% spent no time at all. On the other hand, most (43%) dedicated between 1 and 15 min a week to grooming, 26.3% spent 16–30 min, 18.9% none, 10.1% between 31 and 60 min, and 1.8% more than 60 min. 

### 3.6. Owner Satisfaction with Actual Dog Behaviour

**Figure 1:**
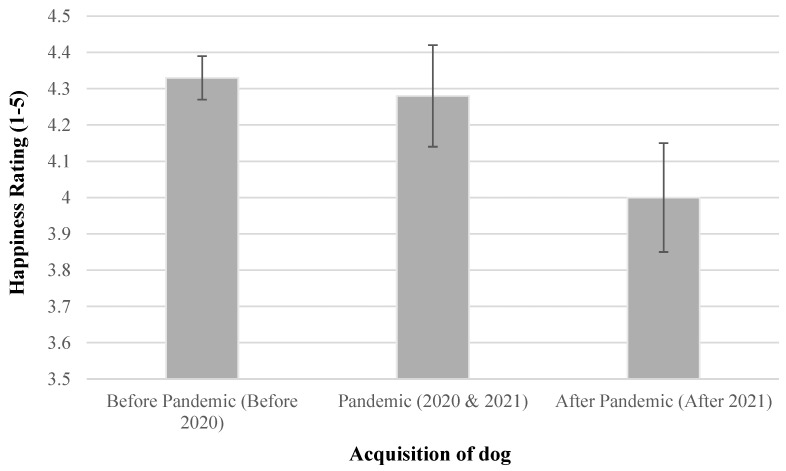
Mean ratings of dog owner happiness with their dog’s behaviour, comparing owners who acquired their dogs before, during, or after the COVID-19 pandemic. Error bars represent the standard error of means.

Participants who currently or previously owned a dog reported that 11% were acquired during the peak pandemic years of 2020 and 2021. Using a non-parametric Kruskal–Wallis test, a significant difference regarding satisfaction with dog behaviour was found among the three groups (*p* = 0.046). Pairwise comparisons found that the significant difference was between the before-pandemic group and the after-pandemic group (*p* = 0.042) and that there was no significant difference between the pandemic group and the before (*p* = 0.695) or after (*p* = 0.380) groups (Figure 1). 

## 4. Discussion

This study aimed to identify the preferred behavioural and physical traits for companion dogs in Australia in 2024, assess if demographics influence these preferences, uncover actual dog characteristics to reference ideal dog traits against, and determine behavioural satisfaction with regard to timing of acquisition—before, during, or after the pandemic. The findings revealed that the ideal Australian canine companion is a healthy, medium-sized dog with a short, low-, or non-shedding coat—a trait popularised on social media as “hypoallergenic”. This dog would ideally be acquired as a puppy through adoption from a shelter or rescue organisation. Additionally, it would cost less than AUD 1000, approximately equating to the median Australian weekly wage of AUD 1300 [34]. Key behavioural traits included being safe with children, housetrained, non-destructive when left alone, and not escaping the property. Most participants wished to spend 31–60 min a day exercising their ideal dog and 1–15 min per week grooming—a time expectation not consistent with highly curated social media images of perfectly groomed low- and non-shedding breeds, which may take hours of grooming to achieve. 

Various associations between demographic factors and ideal dog traits were identified. Female participants strongly preferred de-sexed dogs and valued ‘calm and obedient’ and ‘affectionate and healthy’ behaviours. This stronger preference for a spayed/neutered dog could reflect masculine beliefs and empathy towards the animal by men, who may see their dog as an extension of self [35,36]. Older participants favoured more time exercising and placed greater importance on ‘calm and obedient’ and ‘non-aggressive and safe’ behaviours. These preferences could reflect a slower lifestyle with more time to spend on walks in retirement years. No significant differences between dog owners and non-owners were found following appropriate Bonferroni adjustments. Those with a higher perceived income relative to others were willing to spend more on acquiring their ideal dog, while those who lived in households without children preferred adoption from a shelter or rescue.

The study also gathered information on the characteristics of participants’ previous or current dogs to better understand how these actual traits align with the ideal. This descriptive comparison offers insights into potential gaps between expectations, desires and reality. These discrepancies can highlight areas where expectations may not be met and could inform future education efforts for prospective owners. For example, there was a strong preference for adopting the ideal dog from a shelter or rescue organisation, whereas most participants had acquired their dog from a breeder. This may be because social media is used very effectively to promote ‘rescue’ animals [37], even though the cute puppies and curly-coated breed types made popular through celebrity influencers are less likely to be found among shelter populations in Australia [38]. Additionally, respondents indicated a willingness to invest more money into maintaining and more time exercising their ideal dogs compared to what owners spent on their actual dogs. Participants also showed a slight preference for lower shedding levels than what they experienced with their actual dogs. However, there were no notable differences in breed type (purebred, mixed, or designer), size, acquisition cost, age at acquisition, or grooming requirements. 

Behavioural satisfaction did not differ significantly between those who acquired their dog during the pandemic and those who did not. However, a significant difference was found between pre- and post-pandemic groups, in that those who acquired a dog prior to the pandemic were substantially happier with their dog’s behaviour than those who had acquired their dog following the pandemic. This contradicts findings by Brand and colleagues [10], who reported that almost 97% of owners who purchased their dogs during the pandemic in the United Kingdom reported behavioural issues in their young dogs at 21 months of age. Our findings may result from the post-2021 dogs still being young. Perhaps dogs exhibit more behavioural issues when young [39] that negatively impact owner satisfaction. These may either resolve themselves with age or dogs with undesirable characteristics may be replaced. 

The findings mostly align with previous ideal dog studies conducted more than a decade ago, which reported preferences for medium-sized dogs with short, straight hair, and dogs that were affectionate, friendly, and obedient, safe with children, came when called, were housetrained, and did not escape or destroy property [2,6]. In contrast, however, the present study found no preference regarding coat type, and participants placed less importance on their dog coming when called and showing affection. The first of these may reflect societal/environmental changes that have occurred in the past decade. Many local government areas in Australia now require all dogs to be on a leash at all times, except in enclosed dog parks, which are now more common and may mean that owners place less importance on recall ability. Anecdotally, crating dogs has become more common, reducing opportunities for destructive behaviour, as has the use of dog walkers, dog sitters, and dog daycare centres. Why current owners would place less importance on their dog showing affection is more difficult to explain. While this might reflect owners’ preferring more canine autonomy and less demonstrated affection, it seems more likely a function of different recruitment methods used in the two samples, which are discussed in more detail below. The PCA factors and their means also mirror those found in the original Australian ideal dogs study [2]. The current study found five factors: ‘calm and obedient’ (M = 4.14, SD = 0.49), ‘inhibitory control’ (M = 3.78, SD = 0.65), ‘energy and drive’ (M = 3.08, SD = 0.65), ‘non-aggressive and safe’ (M = 4.22, SD = 0.55), and ‘affectionate and healthy’ (M = 4.32, SD = 0.50). These correspond closely to those identified by King and colleagues: ‘calm/compliant’ (M = 4.00, SD = 0.49), ‘socially acceptable’ (M = 3.68, SD = 0.73), ‘energetic/faithful/protective’ (M = 3.14, SD = 0.54), ‘non-aggressive’ (M = 4.10, SD = 0.63), and ‘sociable/healthy’ (M = 4.36, SD = 0.47). While the specific items loading onto each factor differed, overall, the similarities in the factors and their relative mean scores suggest that, in contrast to what was anticipated, Australians’ preferences for the ideal dog’s physical and behavioural characteristics have remained relatively stable despite societal changes. 

The consistency between Australian studies is striking given demographic differences between the samples. Data collection was separated by 15 years and used different recruitment methods—the opportunistic sample used in the previous study [2] likely attracted dog enthusiasts, whereas the use of Prolific Academic Ltd. in the current study was expected to lead to a less dog-centric sample. Consistent with this is the finding discussed above, that the current sample placed less importance on their dogs showing affection. Indeed, the previous study included a much higher percentage of dog owners (72.3%) compared to the current study (33.8%). This may partially reflect the current cost-of-living crisis and increased work demands, although given that there is no indication that rates of dog ownership are decreasing in Australia, it more likely reflects the different recruitment strategies. Also consistent with this, the gender breakdown in the current study was more balanced (females = 52.9% in the current study versus 79.8% in the previous one) and included 3.6% of participants who identified as non-binary, third gender, self-described, or preferred not to disclose—a range of options that were absent in the original survey. Age ranges were similar between the two studies (18 to 84 years), but King and colleagues reported a higher percentage of respondents whose highest completed education level was secondary school (46.5%) compared to the current study (17.8%), where higher education levels prevailed. Both studies had high proportions of participants without children in their households, 73.2% in the previous study and 70.3% in the current one.

### 4.1. Implications

These insights have practical implications for breeders and prospective dog owners. Breeders can use these data to align their breeding practices with what Australians want in their companion dogs, selectively breeding for preferred temperaments and qualities. The findings can also inform prospective owners by highlighting the importance of selecting dogs whose traits and requirements align with their lifestyle. Readjusting expectations so that they are more realistic, in order to improve the human–dog relationship, may be required to benefit human and animal wellbeing. The lack of significant behavioural dissatisfaction among owners of dogs acquired during the pandemic suggests that the current influx of adult dogs into shelters may have less to do with their behaviours than with other factors, such as post-pandemic lifestyle changes or the current cost-of-living crisis [40]. Strategies to keep these dogs in their current homes are important, particularly since the project aligns with previous findings in showing a strong preference for acquiring dogs as puppies. 

Unlike the previous Australian study by King et al. [2], which did not explore why puppies were preferred, the current study found that the bonding potential, cuteness, trainability, and having more time with the dog were the main reasons for this preference. A preference for puppies aligns with Attachment Theory [31,32]. Many owners who keep dogs as companions refer to them as part of the family, as their child or ‘fur baby’ [41,42], suggesting that many view their dogs as surrogate children. Additional research suggests that the human–dog relationship can resemble that of a human caregiver–infant relationship [32], whereby the four key features of attachment (proximity maintenance/seeking, secure base, safe haven, and separation distress) are observed [43,44] and whereby owners assume the role of caregiver and care for the dog [45]. By acquiring a dog as a puppy, owners may perceive that they can better establish an attachment relationship and ‘raise’ their dog, much like one would raise a child. In addition, households with children were less inclined to adopt from shelters and rescues, a finding consistent with other research [46]. This suggests that shelters and rescues may need to better address misconceptions about the suitability of adult dogs for family homes and the ability of adult dogs to enter into attachment relationships with new owners, although longitudinal research tracking owner satisfaction with dogs purchased from different sources is to be encouraged.

### 4.2. Limitations and Future Directions

Research in this area has predominantly been conducted using convenience samples, often strongly biased towards participants who identify as female and who presumably are very interested in companion dogs. The use of Prolific Academic Ltd. allowed for a more representative sample than previous studies, with many more males participating than would typically be expected. However, the limitations of this study included a higher proportion of non-pet and non-dog owners than expected based on population data [13] and an over-representation of individuals with a tertiary education [47], participants without household children [48], and those who were located in urban areas [49]. This demonstrates that the findings may not fully represent the Australian population but may reflect these groups and their likeliness to be registered with Prolific and complete online questionnaires. Additionally, some important questions were omitted from the questionnaire due to resource constraints, such as social media usage, intention to acquire a dog, preferences for snout length given the surge in brachycephalic popularity, and behaviours of participants’ actual dogs. Addressing these areas will provide a more comprehensive understanding of influences and preferences. 

Future research should aim to engage underrepresented groups for more generalisable findings. Exploring factors such as social media influence, interest in acquiring a dog, perceptions of snout length, and behaviours of actual dogs would be valuable. Moreover, using open-ended questions to examine specific reasons for preferences could benefit rescue organisations and breeders. It will also be important to investigate potential explanations for the difference in owner satisfaction with dog behaviour between pre- and post-pandemic dog-acquisition groups. As discussed previously, dog age may be a factor in this finding, with younger dogs being more likely to display behaviours that decrease owner satisfaction. Many other factors may also contribute to this unexpected finding, however, requiring a longitudinal prospective research design to investigate more fully. It will also be important to establish why families with children are less inclined to adopt from a shelter, potentially exploring beliefs about behavioural challenges or the time required to acclimate a dog to an environment with children. It would be helpful to know if families who purchase from breeders are subsequently more or less satisfied with their dog than those who purchase from shelters. Addressing these gaps could inform strategies for shelters and improve dog adoption rates. 

## 5. Conclusions

This study determined the characteristics of the ideal Australian companion dog in 2024. The ideal dog was identified as medium-sized, with a short, low-, or non-shedding coat, and acquired as a puppy from a shelter or rescue organisation. Ideal behavioural traits included being affectionate and healthy, non-aggressive and safe with children, housetrained, non-destructive when left alone, and not inclined to escape the property. These findings align with previous research and suggest that ideal traits for companion dogs in Australia are relatively stable. Unexpectedly, we found no differences in owner satisfaction regarding the behaviour of dogs acquired during the recent pandemic. This suggests that these dogs are being relinquished to shelters for reasons unrelated to their behaviour, such as post-pandemic lifestyle changes or economic pressures. This information can help policymakers, breeders, and prospective owners, helping to reduce relinquishment and enhance human–dog relationships and overall wellbeing. 

## Figures and Tables

**Table 1:** Sample demographics.

Demographics ^1^		*n*	%
Gender	Male	145	43.5
	Female	176	52.9
	Non-binary/third-gender/self-describe/rather not say	12	3.6
Age (years)	18–24	61	18.1
	25–34	122	36.2
	35–44	88	26.1
	45–54	39	11.6
	55–64	20	5.9
	65 and over	7	2.1
Country of birth	Australia	238	74.6
	Not Australia	81	25.4
Highest education level completed	Year/Grade 10 or below	5	1.5
	Year/Grade 11 or 12	60	17.8
	Certificate, Diploma, Advanced Diploma, Associate degree, technical/trade qualification, TAFE/Polytechnic	62	18.4
	Undergraduate	131	38.9
	Postgraduate	79	23.4
Residence location	Urban	86	25.6
	Suburban (over 10 km from the inner city)	181	53.9
	Regional city (population 50,000 or more)	32	9.5
	Country town/island (population less than 50,000)	29	8.6
	Rural (not in a city or town)	8	2.4
Number of adults per household	1	60	17.9
	2	155	46.3
	3	61	18.2
	4	37	11
	More than 4	22	6.6
Number of children per household	0	237	70.3
	1	55	16.3
	2	34	10.1
	3	9	2.7
	Four or more	2	0.6
Dog ownership	Currently own at least one dog	114	33.8
	Previously owned at least one dog	121	35.9
	Never owned a dog	102	30.3

^1^. Demographic totals may differ from the overall sample size due to non-responses.

**Table 2:** Factor loadings and descriptive statistics for the behavioural subscales derived from a Principal Component Analysis of questionnaire items asking participants to rate the importance of certain behaviours in the ‘ideal dog’.

Calm and Obedient		Inhibitory Control		Energy and Drive		Non-Aggressive and Safe		Affectionate and Healthy	
Items and Factor Loadings		Items and Factor Loadings		Items and Factor Loadings		Items and Factor Loadings		Items and Factor Loadings	
Travels calmly and quietly in the car	0.74	Does not eat his/her own faeces	0.88	Enjoys a lot of exercise	0.78	Does not growl at strangers in public areas	0.81	Enjoys being cuddled and hugged	0.82
Walks calmly without pulling on the leash	0.74	Does not eat other animals’ faeces	0.85	Has a high energy level	0.77	Does not bark at strangers in public areas	0.79	Enjoys being petted	0.77
Comes when he/she is called	0.66	Does not scavenge things found on the street	0.64	Likes to play rough-and-tumble games	0.70	Does not fight with other dogs	0.53	Shows affection towards me	0.70
Lets me groom him/her easily	0.64	Does not dig inappropriately	0.52	Has hunting capabilities (inc. pest control)	0.52	Does not chase wildlife or farm animals	0.37	Lives until he/she is at least 10 years old	0.45
Is confident in new surroundings	0.62	Is not overly excitable	0.47	Is constantly attentive to me	0.51	Is safe with children	0.36	Is physically healthy	0.35
Walks calmly on a leash	0.58	Does not exhibit inappropriate sexual behaviours	0.46	Is protective of myself and my family	0.49				
Is not destructive when left alone for long periods	0.54	Does not beg for food	0.36	Enjoys obedience training	0.42				
Behaves calmly most of the time	0.44								
% variance explained	12.69		11.49		9.69		8.71		7.93
Maximum score	5		5		4.86		5		5
Minimum score	2.5		1.43		1.29		2.60		2.6
Mean	4.14		3.78		3.08		4.22		4.32
Standard deviation	0.49		0.65		0.65		0.55		0.50
Cronbach’s α	0.82		0.83		0.79		0.71		0.70

**Table 3:** Spearman’s correlations between demographics and ‘ideal dog’ characteristics.

Ideal Dog Factor	Age	Income	Number of Children in the Household
Size	−0.12 (335)	−0.00 (334)	0.02 (335)
Shedding level	−0.00 (333)	−0.07 (332)	0.06 (293)
Cost to acquire	−0.09 (334)	0.20 ** (333)	0.12 (334)
Time spent exercising	0.21 ** (335)	0.02 (334)	−0.03 (335)
Time spent grooming	0.03 (335)	0.02 (334)	−0.11 (335)
‘Calm and obedient’	0.20 ** (324)	−0.06 (323)	0.01 (324)
‘Inhibitory control’	0.12 (331)	0.01 (330)	−0.02 (331)
‘Energy and drive’	−0.09 (333)	0.12 (332)	0.11 (333)
‘Non-aggressive and safe’	0.21 ** (325)	−0.06 (324)	−0.02 (325)
‘Affectionate and healthy’	0.03 (330)	0.03 (329)	−0.01 (330)

** *p* < 0.00167 (adjusted as per Bonferroni calculation).

## Data Availability

The data presented in this study are available on request from the corresponding author, provided that approval to release the data is obtained from the La Trobe University Human Ethics Committee.

## Supplementary Materials

The following supporting information can be downloaded at: https://www.mdpi.com/article/10.3390/ani14243627/s1, File S1: Questionnaire; File S2: Supplementary Data Tables; File S3: Original study questionnaire, King et al. [2].

## References

[1] Janssens L., Giemsch L., Schmitz R., Street M., Van Dongen S., Crombé P. (2018). A New Look at an Old Dog: Bonn-Oberkassel Reconsidered. J. Archaeol. Sci..

[2] King T., Marston L.C., Bennett P.C. (2009). Describing the Ideal Australian Companion Dog. Appl. Anim. Behav. Sci..

[3] Wang S.-Z., Yan Y., Widlund M., Qian C.-C., Zhang L.-L., Zhang S.-J., Li Z.-M., Cao P., Dai Q.-Y., Feng X.-T. (2024). Historic Dog Furs Unravel the Origin and Artificial Selection of Modern Nordic Lapphund and Elkhound Dog Breeds. Mol. Biol. Evol..

[4] Eken Asp H., Fikse W.F., Nilsson K., Strandberg E. (2015). Breed Differences in Everyday Behaviour of Dogs. Appl. Anim. Behav. Sci..

[5] Meyer I., Forkman B. (2014). Dog and Owner Characteristics Affecting the Dog–Owner Relationship. J. Vet. Behav..

[6] Diverio S., Boccini B., Menchetti L., Bennett P.C. (2016). The Italian Perception of the Ideal Companion Dog. J. Vet. Behav..

[7] Ozcan M., Ekiz B., Ozturk N., Berk H.O.S. (2019). Factors Affecting Turkish Dog Owners’ Breed Choices, and Their Associations with Socio-Demographic and Dog-Related Variables. Anthrozoös.

[8] van Herwijnen I.R.V., Van Der Borg J.A.M., Naguib M., Beerda B. (2018). Dog Ownership Satisfaction Determinants in the Owner-Dog Relationship and the Dog’s Behaviour. PLoS ONE.

[9] Holland K.E. (2019). Acquiring a Pet Dog: A Review of Factors Affecting the Decision-Making of Prospective Dog Owners. Animals.

[10] Brand C.L., O’Neill D.G., Belshaw Z., Dale F.C., Merritt B.L., Clover K.N., Tay M.-X.M., Pegram C.L., Packer R.M.A. (2024). Impacts of Puppy Early Life Experiences, Puppy-Purchasing Practices, and Owner Characteristics on Owner-Reported Problem Behaviours in a UK Pandemic Puppies Cohort at 21 Months of Age. Animals.

[11] Anderson K.L., Holland K.E., Casey R.A., Cooper B., Christley R.M. (2024). Owner Expectations and Surprises of Dog Ownership Experiences in the United Kingdom. Front. Vet. Sci..

[12] Animal Welfare Victoria (2023). Victorian Pet Census: Survey Findings Report.

[13] Animal Medicines Australia (2021). Pets and the Pandemic: A Social Research Snapshot of Pets and People in the COVID-19 Era.

[14] Anderson C. (2021). Investigating Media Influence on Canine Breed Popularity and Increasing Prevalence of Genetic Linked Disorders. Unpublished Honors Thesis.

[15] Hadnagy N. Compare the Market Reveals the Most Common Cat and Dog Breeds for 2023. https://www.comparethemarket.com.au/news/compare-the-market-reveals-the-most-common-cat-and-dog-breeds-for-2023/..

[16] Petbarn. *Petbarn Pet Pulse July 2022*. https://www.petbarn.com.au/petspot/app/uploads/2011/07/Pet-Pulse-Whitepaper.pdf?utm_campaign=petpulse.

[17] Haenlein M., Anadol E., Farnsworth T., Hugo H., Hunichen J., Welte D. (2020). Navigating the New Era of Influencer Marketing: How to be Successful on Instagram, TikTok, & Co. Calif. Manag. Rev..

[18] Maddox J. (2021). The Secret Life of Pet Instagram Accounts: Joy, Resistance, and Commodification in the Internet’s Cute Economy. New Media Soc..

[19] Di Cioccio M., Pozharliev R., De Angelis M. (2024). Pawsitively powerful: Why and When Pet Influencers Boost Social Media Effectiveness. Psychol. Mark..

[20] Baumeister R.F., Leary M.R. (1995). The Need to Belong: Desire for Interpersonal Attachments as a Fundamental Human Motivation. Psychol. Bull..

[21] Herzog H. (2006). Forty-Two Thousand and One Dalmatians: Fads, Social Contagion, and Dog Breed Popularity. Soc. Anim..

[22] Maclennan T., Smith D. (2019). The Influence of Media on the Ownership of Brachycephalic Breed Dogs. Vet. Nurs. J..

[23] Ghirlanda S., Acerbi A., Herzog H. (2014). Dog Movie Stars and Dog Breed Popularity: A Case Study in Media Influence on Choice. PLoS ONE.

[24] Archer J., Monton S. (2011). Preferences for Infant Facial Features in Pet Dogs and Cats. Ethology.

[25] Ghirlanda S., Acerbi A., Herzog H., Serpell J.A. (2013). Fashion vs. Function in Cultural Evolution: The Case of Dog Breed Popularity. PLoS ONE.

[26] Packer R.M.A., Brand C.L., Belshaw Z., Pegram C.L., Stevens K.B., O’Neill D.G. (2021). Pandemic Puppies: Characterising Motivations and Behaviours of UK Owners Who Purchased Puppies during the 2020 COVID-19 Pandemic. Animals.

[27] Loftus L. (2023). Post COVID-19 Behaviour Problems: It’s Not All about Pandemic Puppies!. In Pract..

[28] Jacobs J., Reese L.A. (2021). Compassion Fatigue Among Animal Shelter Volunteers: Examining Personal and Organizational Risk Factors. Anthrozoös.

[29] The Kennel Club The COVID-19 Puppy Boom—One in Four Admit Impulse Buying a Pandemic Puppy. https://www.thekennelclub.org.uk/media-centre/2020/august/the-covid-19-puppy-boom-one-in-four-admit-impulse-buying-a-pandemic-puppy.

[30] Fraser M.M. (2024). Dog Politics: Species Stories and the Animal Sciences.

[31] Archer J. (1997). Why Do People Love Their Pets?. Evol. Hum. Behav..

[32] Payne E., Bennett P., McGreevy P. (2015). Current Perspectives on Attachment and Bonding in the Dog-Human Dyad. Psychol. Res. Behav. Manag..

[33] Pallant J. (2020). SPSS Survival Manual: A Step by Step Guide to Data Analysis Using IBM SPSS.

[34] Australian Bureau of Statistics (2024). Employee Earnings and Hours, Australia.

[35] Hills A.M. (1993). The Motivational Bases of Attitudes Toward Animals. Soc. Anim..

[36] Belk R.W. (1996). Metaphoric Relationships with Pets. Soc. Anim..

[37] Lee J. (2024). “Cute Saves the World!”: Animal Internet Celebrities’ cute activism in the #adoptdon’tshop Animal Rescue Campaign on Social Media. Soc. Mov. Stud..

[38] Hemy M., Rand J., Morton J., Paterson M. (2017). Characteristics and Outcomes of Dogs Admitted into Queensland RSPCA Shelters. Animals.

[39] Lund J.D., Agger J.F., Vestergaard K.S. (1996). Reported Behaviour Problems in Pet Dogs in Denmark: Age Distribution and Influence of Breed and Gender. Prev. Vet. Med..

[40] Packer R.M.A., Brand C.L., Belshaw Z., Pegram C.L., Dale F., Stevens K.B., O’Neill D.G. (2023). Is UK Puppy Purchasing Suffering a Long COVID Effect? Ongoing Negative Impacts of the COVID-19 Pandemic upon Puppy Purchase Motivations and Behaviours in 2021. Animals.

[41] Animal Medicines Australia (2019). Pets in Australia: A National Survey of Pets and People.

[42] Kwong M.J., Bartholomew K. (2011). “Not Just a Dog”: An Attachment Perspective on Relationships with Assistance Dogs. Attach. Hum. Dev..

[43] Kurdek L.A. (2008). Pet Dogs as Attachment Figures. J. Soc. Pers. Relatsh..

[44] Meredith P., Strong J., Condon L., Lindstrom D., Hill J. (2023). Understanding the Occupational Role of Dog Ownership through the Lens of Attachment Theory: A Survey Study. Br. J. Occup. Ther..

[45] Savalli C., Mariti C. (2020). Would the Dog Be a Person’s Child or Best Friend? Revisiting the Dog-Tutor Attachment. Front. Psychol..

[46] Udvarhelyi-Tóth K.M., Iotchev I.B., Kubinyi E., Turcsán B. (2024). Why Do People Choose a Particular Dog? A Mixed-Methods Analysis of Factors Owners Consider Important When Acquiring a Dog, on a Convenience Sample of Austrian Pet Dog Owners. Animals.

[47] Australian Bureau of Statistics (2023). Education and Work, Australia.

[48] Australian Bureau of Statistics (2022). Households and Families in Australia: Census 2021.

[49] Australian Bureau of Statistics (2022). Location: Census, 2021.

