# Supplementary material for: The Ideal Canine Companion: Re-Exploring Australian Perspectives on Ideal Characteristics for Companion Dogs
Source: Animals (Basel). 2024 Dec 16;14(24):3627. doi: 10.3390/ani14243627 (PMC11672757; doi:10.3390/ani14243627)
Supplement: Supplementary file 1 [file animals-14-03627-s001.zip › File S1.pdf]

File S1: Questionnaire

MONASH University

Characteristics of the ideal companion dog

The purpose of this questionnaire is to gain insight into what characteristics the ideal pet dog should possess. Please answer the following questions about YOUR ideal pet dog as accurately as possible.

In order to participate, you must be aged 18 years of age or over. No personally identifying data are collected in this survey. Participation is completely voluntary and you do not have to answer any question that you do not wish to. However, this research will benefit greatly from your full participation.

SECTION A - Characteristics of your ideal dog

(please tick the appropriate box)

A1. My ideal dog would be:
- ❑ Male
- ❑ Female
- ❑ Sex of the dog is not important

A2. My ideal dog would be:
- ❑ De-sexed (neutered)
- ❑ Not de-sexed
- ❑ Desexing status not important

A3. My ideal dog would have:
- ❑ No hair/fur (eg Chinese Crested Dog)
- ❑ Short straight hair/fur (eg Dalmation)
- ❑ Short curly hair/fur (eg Curly Coated Retriever)
- ❑ Short wiry hair/fur (eg Border Terrier)
- ❑ Medium length straight hair/fur (eg Cocker Spaniel)
- ❑ Medium length curly hair/fur (eg Poodle)
- ❑ Medium length thick hair/fur (eg German Shepherd)
- ❑ Long straight hair/fur (eg Afghan)
- ❑ Long curly hair/fur (eg Hungarian Puli)
- ❑ Long thick hair/fur(eg Alaskan Malamute)
- ❑ Length and type of hair not important

A4. My ideal dog would be:
- ❑ Black
- ❑ White
- ❑ Brown
- ❑ Multi-coloured
- ❑ Other. Please specify _______________
- ❑ Colour not important

A5. My ideal dog would be:
- ❑ Tiny (0-3 kg)
- ❑ Small (4-10kg)
- ❑ Medium 10-20kg)
- ❑ Large (20-40kg)
- ❑ X-Large (40+kg)
- ❑ Size not important

A6. My ideal dog would be a:
- ❑ Pure breed
- ❑ Mixed breed
- ❑ Designer dog
- ❑ Type not important

A7. My ideal dog would be:
- ❑ Acquired as a puppy
- ❑ Acquired as an adult
- ❑ Age of acquisition not important

A8. To maintain my ideal dog, it would cost ______ per week:
- ❑ $0-10
- ❑ $11-20
- ❑ $21-30
- ❑ $31 +
- ❑ Cost not important

A9. My ideal dog would require _____minutes of exercise per day:
- ❑ 0
- ❑ 1-15
- ❑ 16-30
- ❑ 31-60
- ❑ 61+

A10. My ideal dog would require _____minutes of grooming per week:
- ❑ 0
- ❑ 1-15
- ❑ 16-30
- ❑ 31-60
- ❑ 61+

SECTION B

Please rate the importance of each of the following statements by circling the most appropriate response. Your responses should represent your opinion about YOUR ideal pet dog.

1= Extremely unimportant
2= Unimportant
3 = Neither important nor unimportant
4= Important
5= Extremely important

MY IDEAL DOG:

	Extremely unimportant				Extremely important
B1. Is fully housetrained (never soils in house)	1	2	3	4	5
B2. Does not bark inappropriately	1	2	3	4	5
B3. Never jumps on people	1	2	3	4	5
B4. Comes to me when he/she is called	1	2	3	4	5
B5. Is friendly towards strangers	1	2	3	4	5
B6. Walks calmly without pulling on the leash	1	2	3	4	5
B7. Let's me groom him/her easily	1	2	3	4	5
B8. Allows the vet to examine him/her	1	2	3	4	5
B9. Travels calmly and quietly in the car	1	2	3	4	5
B10. Is not destructive when left alone	1	2	3	4	5
B11. Barks at people who enter my property	1	2	3	4	5
B12. Is confident in new surroundings	1	2	3	4	5
B13. Will bite people on command	1	2	3	4	5
B14. Remains calm during thunderstorms or fireworks	1	2	3	4	5
B15. Does not eat its own faeces	1	2	3	4	5
B16. Does not eat other animals' faeces	1	2	3	4	5
B17. Does not scavenge things found on the street	1	2	3	4	5

MY IDEAL DOG:

	Extremely unimportant				Extremely important
B18. Does not bark at strangers in public areas	1	2	3	4	5
B19. Does not growl at strangers in public areas	1	2	3	4	5
B20. Learns new tasks quickly	1	2	3	4	5
B21. Does not chase wildlife or farm animals	1	2	3	4	5
B22. Has hunting capabilities	1	2	3	4	5
B23. Has a high energy level	1	2	3	4	5
B24. Is safe with children	1	2	3	4	5
B25. Behaves calmly most of the time.	1	2	3	4	5
B26. Does not exhibit inappropriate sexual behaviours	1	2	3	4	5
B27. Is not overly excitable	1	2	3	4	5
B28. Does not dig inappropriately	1	2	3	4	5
B29. Does not beg for food	1	2	3	4	5
B30. Shows affection toward me	1	2	3	4	5
B31. Lives until he/she is at least 10 years old	1	2	3	4	5
B32. Is physically healthy	1	2	3	4	5
B33. Enjoys being petted	1	2	3	4	5
B34. Is friendly with other dogs	1	2	3	4	5
B35. Is protective of myself and my family	1	2	3	4	5
B36. Enjoys large amounts of exercise	1	2	3	4	5
B37. Enjoys obedience training	1	2	3	4	5
B38. Does not escape from my property	1	2	3	4	5
B39. Is physically impressive to look at	1	2	3	4	5
B40. Does not fight with other dogs	1	2	3	4	5
B41. Enjoys being cuddled and hugged	1	2	3	4	5

MY IDEAL DOG:

	Extremely unimportant				Extremely important
B42. Likes to play rough and tumble games	1	2	3	4	5
B43. Tolerates being left alone for long periods	1	2	3	4	5
B44. Is constantly attentive to me	1	2	3	4	5

SECTION C

Please list the five characteristics that would be MOST IMPORTANT in your ideal dog

C1. ____

C2. ____

C3. ____

C4. ____

C5. ____

SECTION D

Please rate the degree to which you agree or disagree with each of the following statements by circling the most appropriate response.

1= Strongly Disagree
2= Disagree
3= Neither agree or disagree
4 = Agree
5= Strongly Agree

	Strongly disagree				Strongly agree
D1. A dog's temperament is influenced more by its genes than by training.	1	2	3	4	5
D2. Dogs should always undertake obedience training	1	2	3	4	5
D3. Owners are responsible for their dogs behaviour	1	2	3	4	5
D4. Some dogs cannot be trained	1	2	3	4	5

SECTION E – A few general questions about you.

E1. What is your age in years? ___________________

E2. What is your gender?
- ❑ Male
- ❑ Female

E3. Were you born in Australia?
- ❑ Yes
- ❑ No (In what country were you born? __________________)

E4. What is the highest level of education that you have reached?
- ❑ Completed primary school
- ❑ Completed part of secondary school
- ❑ Completed secondary school or equivalent
- ❑ Completed undergraduate degree
- ❑ Completed postgraduate degree

E5. How many people live in your household, including yourself?
- ❑ One
- ❑ Two
- ❑ Three
- ❑ Four
- ❑ Five or more

E6. How many children do you have living at home?
- ❑ None (please go to question E8)
- ❑ One
- ❑ Two
- ❑ Three
- ❑ Four
- ❑ Five or more

E7. What ages are your children living in the household (please write)? ________________________

E8. What is your postcode? ________________

E9. Which of the following best describes where you live?
- ❑ Flat/unit
- ❑ House without backyard
- ❑ House with backyard
- ❑ Small property (less than 2 acres)
- ❑ Medium property (2-20 acres)
- ❑ Large property (more than 20 acres)
- ❑ Other. Please specify ____________________

E10. How many dogs do you currently own that live with you?
- ❑ 0
- ❑ 1-3
- ❑ 4-6
- ❑ 7-9
- ❑ 10 or more

E11. How many cats do you currently own that live with you?

- ❑ 0
- ❑ 1-3
- ❑ 4-6
- ❑ 7-9
- ❑ 10 or more

E12. How many dogs have you lived with over your lifetime?

- ❑ 0-2
- ❑ 3-5
- ❑ 6-8
- ❑ 8 or more

E13. Do you think dogs make good pets?

- ❑ Yes
- ❑ No

E14. Do you have any other pets living in your household?

- ❑ Yes
- ❑ No

If you CURRENTLY OWN A DOG please complete the following questions. If not, thank you for taking the time to complete this questionnaire. Please return it to us as soon as you are able.

SECTION F

If you CURRENTLY OWN more than one dog please complete this section of the questionnaire describing the details of your OLDEST dog.

(Please tick the appropriate box)

F1. Are you happy with your dogs' behaviour and temperament?

- ❑ Yes
- ❑ No

F2. My dog is:

- ❑ Male
- ❑ Female

F3. My dog is:

- ❑ De-sexed (neutered)
- ❑ Not de-sexed

F4. My dog has:

- ❑ No hair/fur (eg Chinese Crested Dog)
- ❑ Short straight hair/fur (eg Dalmation)
- ❑ Short curly hair/fur (eg Curly Coated Retriever)
- ❑ Short wiry hair/fur (eg Border Terrier)
- ❑ Medium straight hair/fur (eg Cocker Spaniel)
- ❑ Medium curly hair/fur (eg Poodle)
- ❑ Medium thick hair/fur (eg German Shepherd)
- ❑ Long straight hair/fur (eg Afghan)
- ❑ Long curly hair/fur (eg Hungarian Puli)
- ❑ Long thick hair/fur(eg Alaskan Malamute)

F5. My dog is:

- ❑ Black
- ❑ White
- ❑ Brown
- ❑ Multi-coloured
- ❑ Other. Please specify ______________

F6. My dog is:

- ❑ Tiny (0-3 kg)
- ❑ Small (4-10kg)
- ❑ Medium (10-20kg)
- ❑ Large (20-40kg)
- ❑ X-Large (40+kg)

F7. My dog is a:

- ❑ Pure breed
- ❑ Mixed breed
- ❑ Designer dog

F8. My dog was:

- ❑ Acquired as a puppy
- ❑ Acquired as an adult

F9. My dog costs ______ per week:

- ❑ $0-10
- ❑ $11-20
- ❑ $21-30
- ❑ $31 +

F10. My dog requires _____minutes of exercise per day:

- ❑ 0
- ❑ 1-15
- ❑ 16-30
- ❑ 31-60
- ❑ 61+

F11. My dog requires _____minutes of grooming per week:

- ❑ 0
- ❑ 1-15
- ❑ 16-30
- ❑ 31-60
- ❑ 61+

Thank you for taking the time to complete this questionnaire.
