# Supplementary material for: The Ideal Canine Companion: Re-Exploring Australian Perspectives on Ideal Characteristics for Companion Dogs
Source: Animals (Basel). 2024 Dec 16;14(24):3627. doi: 10.3390/ani14243627 (PMC11672757; doi:10.3390/ani14243627)
Supplement: Supplementary file 1 [file animals-14-03627-s001.zip › File S2.pdf]

File S2: Supplementary Data Tables

Table S1: Physical Characteristics of the Ideal Dog

		Goodness of fit		
**Physical characteristics**	**%**	χ^2	**df**	p
Sex		407	4	< .001
Male	15.7			
Female	20.7			
No preference	63.5			
Sexual status		357	4	< .001
Desexed	79.4			
Intact	2.4			
No preference	18.2			
Breed type		603	4	< .001
Purebred	19.5			
Mix or designer	7.4			
No preference	73			
Coat length		210	4	< .001
None	0.3			
Short	39.8			
Medium	30.3			
Long	2.4			
No preference	27.3			
Coat type		338	4	< .001
Smooth	35.8			
Wavy	9.6			
Curly	2.7			
Corded	0.6			
No preference	51.3			
Shedding level		349	4	< .001
Non-shedding	26.6			
Low	56.7			
Moderate	4.5			
Heavy	0.3			
No preference	11.9			
Size		301	4	< .001
Tiny (less than 3 kg)	2.4			
Small (between 3 and 10 kg)	27.9			
Medium (between 11 and 20 kg)	52.2			
Large (between 21 and 40 kg)	16.6			
Extra-large (more than 40 kg)	0.9			

Table S2: Acquisition and Maintenance of the Ideal Dog

Ideal dog factor	%	Goodness of fit χ^2	df	p
Age at acquisition		155	4	< .001
Adult	8.6			
Puppy	68.2			
No preference	23.1			
Cost to acquire		-	-	-
None	2.7			
Less than $1,000	40.2			
Between $1,000 and $2,000	29.2			
Between $2,000 and $4,000	22.3			
Between $4,000 and $10,000	5.7			
Preference for acquisition from a breeder		-	-	-
Preferred	29.9			
No preference	37.6			
Not preferred	32.5			
Preference for acquisition from a shelter or rescue		67.6	4	< .001
Preferred	57.9			
No preference	23.1			
Not preferred	19			
Cost to maintain (weekly)		280	4	< .001
Under $20	4.2			
$20 to $40	31			
$41 to $80	50			
$81 to $150	13.7			
More than $150	1.2			
Time spent exercising (minutes per day)		-	-	-
1 to 15	4.2			
16 to 30	37.4			
31 to 60	48.1			
More than 60	10.4			
Time spent grooming (minutes per week)		203	4	< .001
None	3.9			
1 to 15	39.8			
16 to 30	36.5			
31 to 60	16.3			
More than 60	13.6			

Table S3: Physical Characteristics and Acquisition and Maintenance of Participants' Current or Previous Dog

Dog factors	%
Sex	
Male	51.9
Female	47.7
Don't remember	0.4
Size	
Tiny	2.6
Small	29.4
Medium	37.4
Large	26.4
Extra-large	4.3
Breed type	
Purebred	49.6
Mixed	44.4
Designer	6
Shedding	
Non-shedding	12.7
Low shedding	44.1
Moderate shedding	32.3
Heavy shedding	10.5
Extremely heavy shedding	0.4
Age at acquisition	
Under 3 months	41.5
Between 3 and 6 months	32.3
Between 7 and 12 months	11.8
Between 13 months and 3 years	7.9
Over 3 years old	6.6
Source of acquisition	
Breeder	35.5
Family member or friend	26.3
Shelter or rescue	19.3
Pet shop	10.5
Other	8.3
Cost to acquire	
None	21.5
Less than $1,000	38.5
Between $1,001 and $2,000	15.4
Between $2,001 and $4,000	16.7
Between $,4001 and $10,000	2.2
Don't remember or didn't know	6.1
Cost to maintain (weekly)	
Under $20	13.6
Between $20 and $40	43.9
Between $41 and $80	33.3
Between $81 and $150	6.6

Table S3: Continued

Dog factors	%
More than $150	2.6
Time spent exercising (minutes per day)	
None	1.3
Between 1 and 15	15.4
Between 16 and 30	42.5
Between 31 and 60	29.8
More than 60	11
Time spent grooming (minutes per week)	
None	18.9
Between 1 and 15	43
Between 16 and 30	26.3
Between 31 and 60	10.1
More than 60	1.8

Table S4: Mann-Whitney U Tests

Demographic	Ideal dog factor	*U*	*Df*	*p*
Gender				
	Shedding	9708	282	0.618
	Size	11040	319	0.023
	Cost to acquire	12558	318	0.868
	Cost to exercise	12413	319	0.646
	Cost to groom	12420	319	0.663
	'Calm and obedient'	9292	308	< .001*
	'Inhibitory control'	10647	315	0.026
	'Energy and drive'	11095	317	0.068
	'Non-aggressive and safe'	9953	309	0.009
	'Affectionate and healthy'	9845	315	0.001*
Ownership				
	Shedding	8951	293	0.320
	Size	10614	335	0.067
	Cost to acquire	10778	334	0.137
	Cost to exercise	9953	335	0.007
	Cost to groom	10238	335	0.024
	'Calm and obedient'	10725	324	0.416
	'Inhibitory control'	10436	331	0.096
	'Energy and drive'	10700	333	0.169
	'Non-aggressive and safe'	11068	325	0.845
	'Affectionate and healthy'	11652	330	0.922

Note. Bonferroni Adjustment $\alpha = 0.005$
