# Supplementary material for: The Ideal Canine Companion: Re-Exploring Australian Perspectives on Ideal Characteristics for Companion Dogs
Source: Animals (Basel). 2024 Dec 16;14(24):3627. doi: 10.3390/ani14243627 (PMC11672757; doi:10.3390/ani14243627)
Supplement: Supplementary file 1 [file animals-14-03627-s001.zip › File S3.pdf]

File S3: Original study questionnaire, King et al. [2]

Characteristics of the Ideal Companion Dog Questionnaire

SECTION A – ACQUISITION OF YOUR IDEAL COMPANION DOG

How much thought would you be likely to put into acquiring your ideal dog?

No thought at all	A small amount of thought	A moderate amount of thought	Quite a bit of thought	An extremely high amount of thought

What is your preference for acquiring your ideal dog from a breeder or private owner?

Strong preference to acquire from a breeder or private owner	Moderate preference to acquire from a breeder or private owner	No preference to acquire from a breeder or private owner	Moderate preference not to acquire from a breeder or private owner	Strong preference not to acquire from a breeder or private owner

What is your preference for acquiring your ideal dog from a shelter or rescue organisation?

Strong preference to acquire from a shelter or rescue organisation	Moderate preference to acquire from a shelter or rescue organisation	No preference to acquire from a shelter or rescue organisation	Moderate preference not to acquire from a shelter or rescue organisation	Strong preference not to acquire from a shelter or rescue organisation

Would your ideal dog most likely be acquired as an adult dog or a puppy?

Strong preference to acquire as a puppy[1]	Moderate preference to acquire as a puppy[2]	No preference to acquire as a puppy or adult dog	Moderate preference to acquire as an adult[3]	Strong preference to acquire as an adult[4]

If 1 or 2: How important are the following reasons for preferring to acquire your ideal dog as a puppy?

	Extremely unimportant	Moderately unimportant	Neither important nor unimportant	Moderately Important	Extremely Important
Cuteness	1	2	3	4	5
More years with dog	1	2	3	4	5
Blank slate/trainability	1	2	3	4	5
Stronger bonding	1	2	3	4	5

Being able to choose a reputable breeder	1	2	3	4	5
Availability of a particular breed	1	2	3	4	5

If 3 or 4 - How important are the following reasons for preferring to acquire your ideal dog as an adult?

	Extremely unimportant	Moderately unimportant	Neither important nor unimportant	Moderately Important	Extremely Important
Altruism (doing a good deed)	1	2	3	4	5
Personality is more visible	1	2	3	4	5
Already has basic training	1	2	3	4	5
Avoiding the puppy stage	1	2	3	4	5
Less expensive than a puppy	1	2	3	4	5

SECTION B: COSTS ASSOCIATED WITH YOUR IDEAL DOG

How much would you expect to pay to acquire your ideal dog?

No cost	Less than $1,000	Between $1,000 and $2,000	Between $2,000 and $4,000	Between $4,000 and $10,000	More than $10,000

How much would you expect to pay to maintain your ideal dog each week (including food, toys, grooming, health insurance, vet bills etc)?

Under $20	Between $20 and $40	Between $41 and $80	Between $81 and $150	More than $150

How many times per year, on average, would you expect your ideal dog to require veterinary care?

No vet visits per year	One vet visit per year	Two vet visits per year	Three to five vet visits per year	More than five vet visits per year

How many hours in the average day would you expect your ideal dog to tolerate spending time alone?

None or less than 1 hour alone per day	Between 1 and 3 hours alone per day	Between 3 and 6 hours alone per day	Between 6 and 12 hours alone per day	More than 12 hours alone per day

How many minutes of exercise per day, on average, would you expect your ideal dog to require?

No minutes of exercise per day	Between 1 and 15 minutes of exercise per day	Between 15 and 30 minutes of exercise per day	Between 30 and 60 minutes of exercise per day	More than 60 minutes of exercise per day

How many minutes of training per week, on average, would you expect your ideal dog to require?

No minutes of training per week	Between 1 and 15 minutes of training per week	Between 15 and 30 minutes of training per week	Between 30 and 60 minutes of training per week	More than 60 minutes of training per week

How many minutes of grooming per week, on average, would you expect your ideal dog to require?

No minutes of grooming per week	Between 1 and 15 minutes of grooming per week	Between 16 and 30 minutes of grooming per week	Between 31 and 60 minutes of grooming per week	More than 60 minutes of grooming per week

SECTION C – BEHAVIOUR OF YOUR IDEAL DOG

Please rate the importance of each of the following characteristics by selecting the most appropriate response. Your response should represent your opinion about your ideal pet dog.

	Extremely unimportant	Moderately unimportant	Neither important nor unimportant	Moderately Important	Extremely Important
My ideal dog is fully housetrained (never soils the house)	1	2	3	4	5
My ideal dog is friendly with other dogs	1	2	3	4	5
My ideal dog does not jump on people	1	2	3	4	5
My ideal dog comes when he/she is called	1	2	3	4	5
My ideal dog is friendly towards strangers	1	2	3	4	5

My ideal dog walks calmly without pulling on the leash	1	2	3	4	5
My ideal dog lets me groom him/her easily	1	2	3	4	5
My ideal dog travels calmly and quietly in the car	1	2	3	4	5
My ideal dog is not destructive when left alone for long periods	1	2	3	4	5
My ideal dog is confident in new surroundings	1	2	3	4	5
My ideal dog will bite people on command	1	2	3	4	5
My ideal dog remains calm during thunderstorms or fireworks	1	2	3	4	5
My ideal dog does not eat his/her own faeces	1	2	3	4	5
My ideal dog does not eat other animals' faeces	1	2	3	4	5
My ideal dog does not scavenge things found on the street	1	2	3	4	5
My ideal dog does not bark at strangers in public areas	1	2	3	4	5
My ideal dog does not growl at strangers in public areas	1	2	3	4	5
My ideal dog learns new tasks quickly	1	2	3	4	5
My ideal dog does not chase wildlife or farm animals	1	2	3	4	5
My ideal dog has hunting capabilities (including pest control)	1	2	3	4	5

My ideal dog has a high energy level	1	2	3	4	5
My ideal dog is safe with children	1	2	3	4	5
My ideal dog behaves calmly most of the time	1	2	3	4	5
My ideal dog does not exhibit inappropriate sexual behaviours	1	2	3	4	5
My ideal dog is not overly excitable	1	2	3	4	5
My ideal dog does not dig inappropriately	1	2	3	4	5
My ideal dog does not beg for food	1	2	3	4	5
My ideal dog shows affection towards me	1	2	3	4	5
My ideal dog lives until he/she is at least 10 years old	1	2	3	4	5
My ideal dog is physically healthy	1	2	3	4	5
My ideal dog enjoys being petted	1	2	3	4	5
My ideal dog walks calmly on leash	1	2	3	4	5
My ideal dog is protective of myself and my family	1	2	3	4	5
My ideal dog enjoys a lot of exercise	1	2	3	4	5
My ideal dog enjoys obedience training	1	2	3	4	5
My ideal dog does not escape from my property	1	2	3	4	5
My ideal dog is physically attractive to look at	1	2	3	4	5
My ideal dog does not fight with other dogs	1	2	3	4	5

My ideal dog enjoys being cuddled and hugged	1	2	3	4	5
My ideal dog likes to play rough and tumble games	1	2	3	4	5
My ideal dog is constantly attentive to me	1	2	3	4	5

SECTION D – PHYSICAL CHARACTERISTICS OF YOUR IDEAL DOG

What is your preference regarding the sex of your ideal dog?

Strong preference for male	Moderate preference for male	No preference for either male or female	Moderate preference for female	Strong preference for female

What is your preference regarding the sexual status of your ideal dog?

Strong preference for dog to be desexed (neutered)	Moderate preference for dog to be desexed (neutered)	No preference for whether dog is desexed or sexually intact	Moderate preference for dog to be sexually intact	Strong preference for dog to be sexually intact

What is your preference regarding the size of your ideal dog?

Tiny (Less than 3 kg)	Small (between 3 and 10 kg)	Medium (between 10 and 20 kg)	Large (between 20 and 40 kg)	Extra large (more than 40 kg)

What is your preference regarding the body type of your ideal dog?

Very fine body type (Whippet & Greyhound)	Moderate body type (Kelpie & Cavoodle)	Medium body type (Labrador Retriever & Beagle)	Solid body type (Staffy & Pug)	Very solid body type (Mastiff & French Bulldog)

What is your preference regarding the coat colour of your ideal dog?

Strong preference for lighter colours	Moderate preference for lighter colours	No colour preferences	Moderate preference for darker colours	Strong preference for darker colours

What is your preference regarding the coat pattern of your ideal dog?

Strong preference for mix of colours	Moderate preference for mix of colours	No preference regarding coat pattern	Moderate preference for single colour	Strong preference for single colour

What is your preference regarding the length of your ideal dog's hair/fur:

No hair/fur[1]	Short hair/fur	Medium hair/fur	Long hair/fur	No preference regarding hair/fur length

If 1, skip the next three questions

What is your preference regarding the texture of your ideal dog's hair/fur?

Smooth coat	Wavy hair/fur	Curly hair/fur	Corded hair/fur	No preference regarding texture of hair/fur

What is your preference regarding how much your dog sheds its hair/fur?

No shedding	Low shedding	Moderate shedding	Heavy shedding	No preference regarding amount of shedding

What is your preference regarding the breed or breed type of your ideal dog?

Strong preference for purebred dog[1]	Moderate preference for purebred dog[2]	No preference regarding breed or breed type[3]	Moderate preference for mixed breed or designer dog[4]	Strong preference for mixed breed or designer dog[5]

* In this question, purebred means a dog with many generations of ancestors belonging to the same breed (eg Labrador Retriever, Chihuahua, Siberian Husky)

If 1 or 2:

Please list your top 5 ideal breeds

Open textbox

____

How important are the following reasons for preferring these breeds?

	Extremely unimportant	Moderately unimportant	Neither important	Moderately Important	Extremely Important

			nor unimportant		
Specific behavioural features	1	2	3	4	5
Specific physical features	1	2	3	4	5
Previous personal experience	1	2	3	4	5
Being able to compete in specific dog sports	1	2	3	4	5

If 4 or 5: Is your preference for a mixed breed with a specific combination of breeds (eg designer breeds like cavoodles, spoodles, pugaliers) or for a mixed breed with unknown parentage (eg mutt)?

Strong preference for designer breed[1]	Moderate preference for designer breed[2]	No preference regarding breed or breed type[3]	Moderate preference for mixed breed[4]	Strong preference for mixed breed[5]

If 1 or 2:

Please list your top 5 ideal designer breeds

Open textbox

____

How important are the following reasons for preferring your ideal dog to be a designer breed?

	Extremely unimportant	Moderately unimportant	Neither important nor unimportant	Moderately Important	Extremely Important
Specific behavioural features	1	2	3	4	5
Specific physical features	1	2	3	4	5
Previous personal experience	1	2	3	4	5
Avoidance of genetic health issues	1	2	3	4	5
Hybrid vigour	1	2	3	4	5

If 4 or 5

How important are the following reasons for preferring your ideal dog to be a mixed breed?

	Extremely unimportant	Moderately unimportant	Neither important nor unimportant	Moderately Important	Extremely Important
Previous personal experience	1	2	3	4	5
Avoidance of genetic health issues	1	2	3	4	5
Hybrid vigour	1	2	3	4	5
Individuality of a specific dog	1	2	3	4	5

SECTION E – CURRENT OR PREVIOUS DOG

Which of the following best describes you?

I currently own more than one dog[1]	I currently own one dog[2]	I do not currently own a dog but have previously owned more than one dog[3]	I do not currently own a dog but have previously owned one dog[4]	I have never owned a dog[5]

What is the name of your current or most recent dog?

If you own or owned more than one dog, choose one to answer the next pages of questions about

Insert dog's name___________

Use [Dogs_Name] to refer to the dog by name in the following questions

How happy overall are/were you with [Dogs_Name]'s behaviour?

Very unhappy	Moderately unhappy	Neither happy nor unhappy	Moderately happy	Very happy

How happy overall are/were you with [Dogs_Name]'s health and longevity?

Very unhappy	Moderately unhappy	Neither happy nor unhappy	Moderately happy	Very happy

What sex is/was [Dogs_Name]

Female	Male	Don't Know

What is/was [Dogs_Name]'s reproductive status?

Desexed	Sexually intact	Don't Know

What size is/was [Dogs_Name]?

Tiny (Less than 3 kg)	Small (between 3 and 10 kg)	Medium (between 10 and 20 kg)	Large (between 20 and 40 kg)	Extra large (more than 40 kg)

What body type is/was [Dogs_Name]?

Very fine body type (Whippet & Greyhound)	Moderate body type (Kelpie & Cavoodle)	Medium body type (Labrador Retriever & Beagle)	Solid body type (Staffy & Pug)	Very solid body type (Mastiff & French Bulldog)

What label best describes [Dogs_Name]?

Purebred	Designer Breed	Mixed

What breed or mix of breeds best describes [Dogs_Name]?

____

Relative to other dogs, how much did/does [Dogs_Name] shed?

No shedding	Low shedding	Moderate shedding	Heavy shedding	Extremely heavy shedding

How old was [Dogs_Name]? when you acquired them?

Under 3 months of age	Between 3 and 6 months of age	Between 6 and 12 months of age	Between 12 months and 3 years of age	Over 3 years of age

Where did you acquire [Dogs_Name] from?

Breeder	Pet shop	Friend or family member	Shelter or rescue group	Other

When was [Dogs_Name] acquired?

Before 2020	2020 (Early in pandemic)	2021 (Middle of pandemic)	2022 (Late in pandemic)	2023	2024

Before you acquired [Dogs_Name], to what extent were you feeling lonely, distressed or unsafe?

Not at all	A little	A moderate amount	Quite a bit	Very much

To what extent did you feel less lonely, distressed or unsafe after acquiring [Dogs_Name]?

Not at all	A little	A moderate amount	Quite a bit	Very much

How much did [Dogs_Name] cost to acquire?

No cost	Less than $1,000	Between $1,000 and $2,000	Between $2,000 and $4,000	Between $4,000 and $10,000	More than $10,000	Don't know

How much does/did [Dogs_Name] cost to maintain each week (including food, toys, grooming, health insurance, vet bills etc)?

Under $20	Between $20 and $40	Between $40 and $80	Between $80 and $150	More than $150

How many times per year, on average, does/did [Dogs_Name] require veterinary care?

No vet visits per year	One vet visit per year	Two vet visits per year	Three to five vet visits per year	More than five vet visits per year

How many minutes of exercise per day, on average, does/did [Dogs_Name] require?

No minutes of exercise per day	Between 1 and 15 minutes of exercise per day	Between 15 and 30 minutes of exercise per day	Between 30 and 60 minutes of exercise per day	More than 60 minutes of exercise per day

How many minutes of training per week, on average, does/did [Dogs_Name] require?

No minutes of training per week	Between 1 and 15 minutes of training per week	Between 15 and 30 minutes of training per week	Between 30 and 60 minutes of training per week	More than 60 minutes of training per week

How many minutes of grooming per week, on average, does/did [Dogs_Name] require?

No minutes of grooming per week	Between 1 and 15 minutes of grooming per week	Between 15 and 30 minutes of grooming per week	Between 30 and 60 minutes of grooming per week	More than 60 minutes of grooming per week

SECTION F – PARTICIPANT DEMOGRAPHICS

In what year were you born?

Drop down with years.

In what country were you born?

Drop down with countries

If anything other than Australia: For approximately how long have you lived in Australia?

- Less than one year
- Between one and two years
- Between two and five years
- Between five and ten years
- Longer than ten years

Which of the following best describes your gender identity?

- Man
- Woman
- Non-binary/third gender
- Prefer to self-describe ____________________
- I'd rather not say

What is the highest level of education that you have completed?

- No formal schooling
- Year/Grade 10 or below (up to age 16 years)
- Year/Grade 11 or 12 (above age 16 years)
- Certificate, diploma, advanced diploma, associate degree, technical/trade qualification, TAFE/Polytechnic
- Undergraduate University (Bachelor's degree)
- Postgraduate University (Master's degree/PhD)
- Other. Please specify _____

Approximately how many hours do you spend in paid work per week?

- None (I am unemployed, retired or engaged in unpaid caregiver work)
- Fewer than 10 hours
- Between 10 and 20 hours
- Between 20 and 30 hours

- More than 30 hours

On an average weekday, for how many hours is no one at home?

Sliding scale, from 0-24

Which of the following best describes your household income, relative to others in your community?

- Very below average
- Moderately below average
- Average
- Moderately above average
- Very above average

How many adults, over 18 years of age and including yourself, typically reside in your household?

- 1
- 2
- 3
- 4
- More than 4

How many children, under 18 years of age, typically reside in your household?

- None (*skip next question*)
- 1
- 2
- 3
- 4+

Please indicate the age(s) of the child/children in your home. Please select all that apply.

- Less than one year
- 1-4 years
- 5-10 years
- 11-15 years
- 15-17 years

What is your postcode? _________

Which of the following best describes where you live?

- Urban (inner city)
- Suburban (over 10 kilometres/6 miles from the inner city)
- Regional city (population 50,000 or more)
- Country town/island (population less than 50,000)
- Rural (not in a city or a town)

Which of the following best describes your place of residence?

- Apartment
- Townhouse/unit
- House

- Other. Please specify ___________

Does your residence have an outdoor area (e.g. balcony, courtyard, backyard)?

- Yes
- No (*skip next question*)

Which of the following best describes your residence's outdoor area?

- Balcony
- Small backyard/courtyard
- Medium to large backyard/courtyard
- Acreage or farmstead property
- Other. Please specify ______

How many dogs do you currently own that live with you?

- 0
- 1-3
- 4-6
- 7-9
- Ten or more

How many pets other than dogs do you currently own that live with you?

- 0
- 1-3
- 4-6
- 7-9
- Ten or more

How many dogs have you lived with over your lifetime?

- 0
- 1-3
- 4-6
- 7-9
- Ten or more
